# Finding and Producing Probiotic Glycosylases for the Biocatalysis of Ginsenosides: A Mini Review

**DOI:** 10.3390/molecules21050645

**Published:** 2016-05-16

**Authors:** Seockmo Ku

**Affiliations:** Laboratory of Renewable Resources Engineering, Department of Agricultural and Biological Engineering, Purdue University, West Lafayette, IN 47907-2022, USA; sku@purdue.edu; Tel.: +1-765-637-1975

**Keywords:** ginsenoside, probiotics, enzyme conversion, biocatalysis, whole cell conversion, enzyme preparation

## Abstract

Various microorganisms have been widely applied in nutraceutical industries for the processing of phytochemical conversion. Specifically, in the Asian food industry and academia, notable attention is paid to the biocatalytic process of ginsenosides (ginseng saponins) using probiotic bacteria that produce high levels of glycosyl-hydrolases. Multiple groups have conducted experiments in order to determine the best conditions to produce more active and stable enzymes, which can be applicable to produce diverse types of ginsenosides for commercial applications. In this sense, there are various reviews that cover the biofunctional effects of multiple types of ginsenosides and the pathways of ginsenoside deglycosylation. However, little work has been published on the production methods of probiotic enzymes, which is a critical component of ginsenoside processing. This review aims to investigate current preparation methods, results on the discovery of new glycosylases, the application potential of probiotic enzymes and their use for biocatalysis of ginsenosides in the nutraceutical industry.

## 1. Ginsenosides

Ginseng (*Panax ginseng* C.A. Meyer), a popular Asian value-added plant medicine, contains various saponins called ginsenosides, which act as the major functional ingredient. Ginseng products have dominated the nutraceutical market in Asian countries as one of the highest selling functional food products for the last decade [1]. With its beneficial effects, ginseng continues to grow in sales. Various health benefits of ginsenosides have been introduced by multiple researchers; the major functionalities of ginsenosides include: (i) anti-cancer [2]; (ii) immunomodulatory [3]; (iii) anti-obesity [4]; (iv) energy boosting [5]; (v) liver and brain function [6,7]; and (vii) antioxidant effects [8]. Currently, more than 40 kinds of ginsenosides have been reported [1]. Based on the types, number and/or location of the sugars on it, the functional effects and types of ginsenosides are varied [9]. Full chemical names of multiple ginsenosides are shown in Table 1.

Conventionally, ginseng has been consumed orally as a root itself or it can be consumed in energy beverages, teas and functional supplements as a powder or extract. However, the oral ingestion of ginseng is regarded as an ineffective way to absorb major ginsenosides because of: (i) poor gastrointestinal tissue permeability [10]; (ii) low solubility in saliva [11]; (iii) different cleavage rates by stomach acid [12]; and (iv) different types of naturally-occurring microbiota in an individual [13]. According to Hasegawa, the biological usage rates of orally-ingested glycosylated ginsenosides in the intestinal tracks are notably low (about Rb1, 0.1% to 4.4%; Rb2, 3.7%) [14], and these ginsenosides are easily moved out through the biliary or urinary systems [15,16].

## 2. Biocatalytic Process of Ginsenosides Using Probiotic Enzymes

**Figure 1:**
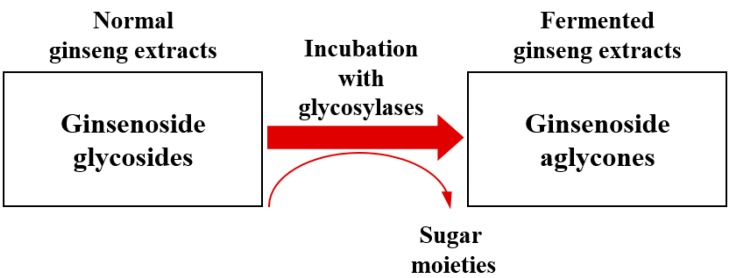
Biocatalysis of ginsenoside glycosides using microbial glycosyl hydrolases.

In terms of designing a biocatalytic process, multiple aspects should be considered: (i) determination of the phytochemicals to be catalyzed; (ii) selection of substrates; (iii) pathways or chemical reactions [19]. For enhanced absorption rates and *in vivo* activities, the structural conversion of glycosylated ginsenosides into aglycone forms has been proposed by several scientific reports; conversion to aglycone forms results in a higher permeability into the human plasma across the intestinal tissues, enabling health-promoting properties [20,21]. Due to the significant functionality of aglycone-type ginsenosides, various pharmaceutical and nutraceutical companies have attempted to produce ginseng products containing high contents of ginsenoside aglycones [22]. A representation of ginsenoside catalysis to produce ginsenoside aglycones using a microbial enzyme is shown in Figure 1.

Multiple processing methods, including chemical reactions (*i.e*., acid or alkaline cleavages), were developed for the purpose of deglycosylation of ginsenosides [23,24,25]. However, these methods do not selectively hydrolyze sugar moieties on ginsenosides and cause side reactions, including epimerization, hydration and hydroxylation, as well as the formation of by-products [25,26]. In order to address these issues, multiple groups in the food industry and academia have tried to develop novel protocols for effective catalysis using microbial enzymes to convert ginsenoside glycosides into aglycone forms [22]. To synthesize the desired end products, enzymatically-catalyzed ginsenoside conversion is superior to chemically-catalyzed approaches due to high enzymatic selectivity in hydrolyzing the sugar moieties [27]. Unlike ruminants that effectively digest herb-derived components, humans show limited utilization of the phytochemicals in their small intestine; ginsenosides move into the large intestine and structurally degrade to utilizable forms by naturally-occurring microbiota residing in the intestinal lining [28]. Even though gut microorganisms hydrolyze glycosylated ginsenosides in the intestinal track, the clinical efficacy of glycosylated ginsenosides is varied, since the naturally-occurring gut microbiota in the host is different for each individual [29]. Therefore, artificial deglycosylation of the ginsenoside before oral absorption is an effective way to promote the health benefits of ginseng [30].

One of the key characteristics of microorganisms for producing deglycosylated ginsenosides as a food or food ingredient is their GRAS (generally regarded as safe) status. The U.S. Food and Drug Administration (FDA) offers the list of regularly-approved GRAS microorganisms [31]. Specifically, the use of probiotic bacteria and their enzymes has shown profound potential with pragmatic applications in the food industry. According to WHO and FAO, probiotics are defined as “live microorganisms which when administered in adequate amounts confer a health benefit on the host” [32]. Heyman and Ménard explained probiotics as “a live microbial feed supplement which beneficially affects the host by improving its intestinal microbial balance” [33]. Because of functional effects on their hosts, probiotics are commonly used in the functional food and feed industry given their application to human nutraceuticals and animal feeds. Given these beneficial effects, market analysts have estimated the size of the global probiotics market to grow at a compound annual growth rate of 7.7% and to reach USD 52.3 billion by 2020 [34]. Among the various probiotic bacteria, *Lactobacillus* and *Bifidobacterium* spp. are known to synthesize various glycosidases (EC 3.2.1), including β-glucosidase (EC 3.2.1.2), cellulase (EC 3.2.1.4), β-galactosidase (EC 3.2.1.23), α-l-arabinofuranosidase (EC 3.2.1.55), α-l-arabinopyranosidase (no EC number) and β-xylosidase (E.C. 3.2.1.37), which are necessary for the deglycosylation of ginsenosides. These microorganisms are known to have evolved from inside of the host’s intestinal tracks where indigestible dietary fibers are their major carbon sources, producing a variety of glycosidases, which can effectively convert glycosides into aglycones [35].

## 3. Commercial Application

According to Baeg and So, the value of the global ginseng market is worth USD 2.08 billion, with South Korea attributed with over half (1.14 billion USD) of the market value, making it the primary ginseng production county in the world [36]. The use of ginseng and ginsenosides continues to appear in an extending spectrum of markets due to the recognition of potential applications in multiple new industrial sectors (e.g., cosmetics, beverage, nutraceuticals, processed food and liquor). Recently, to dominate the ginseng market, multiple bio-venture businesses and food conglomerates have applied for patents related to biocatalysis of ginsenosides using probiotic enzymes [22]. These popular food conglomerates include: Nongshim, Lotte Chilsung, Chong Kun Dang, Korea Yakult, Daesang and Woongjin Foods. Various probiotic strains have been screened on the basis of their biocatalytic properties for various ginsenosides, as determined by thin-layer chromatography and/or high-performance liquid chromatography analysis. Recently, to be differentiated in the market, various companies have launched and developed fermented ginseng products that use the indigenous functional abilities of the probiotics and the catalytic effects of probiotic enzymes. By combining probiotics and ginsenoside in a single product, companies gain both technical and marketing advantages by denoting the exact concentration of deglycosylated ginsenosides on the package.

## 4. Recombinant Glycosylase Expression in *Escherichia coli*

Current progress in biocatalytic technology focuses on using microbial enzymes to produce various nutraceutical products from ginseng [29,30]. Specifically, probiotic bacteria constitute an important source for enzyme preparation. Microorganisms are easily influenced by environmental conditions and produce a variety of metabolites as a response. As a result, a variety of research groups have conducted experiments to determine the best conditions for microbial enzyme production with commercial applications [27]. To address this, the use of transgenic microorganisms has been proposed to enable the use of additional tools for producing glycosyl hydrolases.

The use of recombinant bacteria is a common trend for ginsenoside conversion due to the significant efficacy of biotransformation via recombinant enzymes. As a result, extensive studies have been carried out to minimize processing time and to reduce cost [37]. Enzyme production processes from microorganisms should achieve a balance between cell biomass and enzyme productivities for biotechnological applications [38]. Normally, in practical industrial applications, the use of aerobic microorganisms is more accepted to reduce cost and facilitate enzyme manufacturing scale-up, since growth rates are faster and enzyme productivity is greater than anaerobic microorganisms. Additionally, culturing of aerobic bacteria is much simpler than that for anaerobic microorganisms, since anaerobic conditions and special microbial media are not necessary [39].

Common microorganisms containing foreign gene expression for the purpose of effective production of enzymes and ginsenoside conversion are *E. coli*. [37]. Specifically, the genome of *E. coli* is very easy to modify due to its simplicity (*i.e*., 4400 genes) and also multiplies exponentially. Under a suitable environment (*i.e*., enriched culture media), the germination time for normal wild-type *E. coli* is less than 30 min. In the biotech domain, these characteristics are highly advantageous [40]. Additional uses include transgenic *E. coli*, which expresses human genes and has been successfully used for human growth hormone and insulin production in the pharmaceutical and medicine manufacturing industry [41].

Many researchers have attempted to structurally transform glycosylated ginsenosides using various recombinant β-glucosidase overexpressed in *E. coli*. For example, ginsenoside Rb1 was converted into compound K by the recombinant β-glucosidase cloned from *Microbacterium esteraromaticum* [42]. Hong *et al.* [43] also used recombinant β-glucosidase from *Flavobacterium johnsoniae* to produce ginsenoside Rd and F2. Cui *et al.* [44] hydrolyzed ginsenoside Re and Rg1 into Rg2 and Rh1 using recombinant β-glucosidase cloned from *Actinosynnema mirum*. Ginsenoside Rb1 and Rd were also structurally transformed into F2 by recombinant β-glucosidase from *Paenibacillus mucilaginosus* [45]. Other glycosyl hydrolases were cloned and overexpressed in addition to β-glucosidase. Recombinant α-arabinofuranosidase and α-arabinopyranosidase from *Bifidobacterium longum* H-1 were applied to hydrolyze ginsenoside Rc and Rb2 [46]. β-Xylosidase cloned from *Bifidobacterium breve* K-110 was used to transform ginsenoside Ra1 into ginsenoside Rb2 [47]. All of the above-mentioned recombinant enzymes were overexpressed in the *E. coli* system.

*E. coli* recombinant enzyme technology will likely be applied to medical or pharmaceutical industries to produce ginsenoside aglycones. However, for the pragmatic application into the nutraceutical and food industry, the use of these bacteria will likely be a sensitive issue in regards to product marketing, since the majority of consumers identify *E. coli* as a common inedible bacteria. Some *Microbacterium* and *Flavobacterium* spp. are also reported as a food, feed and/or water contaminant [43,48,49]. The food market, especially nutraceuticals, is fundamentally different from the biomedical or pharmaceutical market [50].

## 5. Recombinant Glycosylase Expression in Probiotic Bacteria

The use of transformed probiotic bacteria with recombinant plasmids might be an alternative approach to the *E. coli* system in the nutraceutical industry for several reasons: (i) probiotics are appropriate to produce food-grade expression of glycosylases; (ii) the price of commercially-available media (*i.e*., de Man, Rogosa and Sharpe media (MRS)) for probiotics is only three-fold more expensive than culture media for *E. coli*; (iii) probiotic bacteria have been considered as GRAS in multiple food products already [51,52]. However, only a few cases using genetically-engineered probiotics have been applied to biotransformation of ginsenosides. For instance, Youn *et al.* [53] have characterized the recombinant β-glucosidase cloned from *Bifidobacterium animalis* and expressed in *Bifidobacterium bifidum* BGN4 to produce ginsenoside aglycones from ginsenoside Rb1 and Rb2. This enzyme showed a broad substrate specificity towards phytochemicals (*i.e*., isoflavones, quercetins and disaccharides) with enhanced enzyme activity compared to those of its unaltered strain. Li *et al.* [54] discussed the biocatalysis of ginsenoside Rb1 and Rd into F2 by recombinant β-glucosidase expressed in *Lactococcus lactis* NZ9000 and cloned from *Paenibacillus mucilaginosus*. They evaluated the molecular mass of purified β-glucosidase and the resultant recovery percentage of ginsenoside F2. The authors highlighted codon optimization to reduce unfavorable codons, resulting in effective β-glucosidase production.

However, the application of recombinant probiotic bacteria in the nutraceutical industry has struggled due to consumer’s substantial resistance. In the market, food customers continue to show negative responses with respect to the use of GM organisms in foods. According to recent reports, 70% of respondents showed significant awareness of GM organisms in their food, and 92% of respondents stated that food packaging should require notification of the use of GM organisms [55,56,57]. Because of many factors, the application of GM organisms as a major or minor ingredient in food has become a significant barrier for food manufacturers and marketers alike [57,58].

## 6. Classical Probiotic Enzyme Preparation

To overcome the barrier of GM and/or non-edible bacteria controversies, multiple groups are looking for novel probiotic strains and cell culture conditions to produce glycoside hydrolases using classical enzyme production methods. Classical methods generally depend on the indigenous properties of probiotic bacteria after downstream processing (e.g., purified enzyme, cell-free extracts and disrupted cell suspensions) or as it is (e.g., whole cell). In terms of designing a biotransformation process of ginsenoside using probiotic enzymes, an important consideration is the discovery of an appropriate probiotic strain or enzyme using screening processes. One of the fundamental methods for screening glycoside hydrolase-producing microorganisms is the use of physical (e.g., homogenizing, sonication, bead-beating and cell disruptor), chemical (cell lysis cocktail) or non-chemical (*i.e*., endopeptidase, carboxypeptidases, lysozymes and lytic transglycosylases) breakage methods for releasing cytosolic enzymes. Because of the presence of biological barriers, such as cell membranes, some glycosides have shown a limited penetration ratio, resulting in the restriction of desirable catalytic reactions [54]. By mixing the collected enzymes from disrupted bacteria with artificial substrates, such as *p*-nitrophenyl glycosides, the physicochemical characterization of the microbial enzyme can be examined by evaluating the released *p*-nitrophenol levels.

For example, Chi *et al.* [17,18,59] used cell-free extracts obtained from multiple probiotic strains for ginsenoside biocatalysis. When using intracellular enzymes, it is critical for ginsenoside glycosides to permeate the microbial cell wall and membrane effectively. To accomplish this, cell disruption or sonication processes were used to open and degrade outer cell membranes for the purpose of extracting intracellular glycosyl hydrolases. Using the centrifugation step, they removed cell debris intentionally and then only used supernatants as crude microbial enzymes.

In their work, through *Bifidobacterium* spp. Int57 and *Bifidobacterium* spp. SJ32, ginsenoside Rb2 was transformed into compound K via ginsenoside Rd and F2. The crude enzyme extracted from *Bifidobacterium* spp. Int57 also catalyzes Rb2 into Rd or F2, while it catalyzes Rb1 into Rd, F2 and compound K [17,59]. *Lactobacillus delbrueckii* was used to produce ginsenoside Rh2 from Rb2. *Bifidobacterium* sp. SH5 enzyme successfully transformed Rb2 into F2 via Rd. Both *Leuconostoc paramesenteroides* and *Lactobacillus delbrueckii* transformed ginsenoside Rb1 and produced F2 and Rh2, respectively. *Bifidobacterium* spp. Int57, *Bifidobacterium* spp. SJ32, *Lactobacillus delbrueckii* and *Leuconostoc paramesenteroides* transformed ginsenoside Rb1 into Rh2 via Rd and F2. *Bifidobacterium* sp. SH5 hydrolyzed ginsenoside Rb1 to F2. Ginsenoside Re was converted to Rh1 using *Bifidobacterium* spp. Int57 and *Bifidobacterium* spp. SJ32 [17,18].

Quan *et al.* [60,61,62] employed different approaches to screen enzyme-producing probiotic bacteria. They first screened naturally-occurring lactic acid microbiota from Korean Kimchi and then carried out colorimetric assays to isolate *Lactobacillus* and *Leuconostoc* spp., which produce extracellular-β-glucosidase. Because all of the screened cells produced exoenzymes, they simply used protein precipitation to collect enzymes from media instead of physical or chemical cell disruption processes. The screened *Leuconostoc citreum* LH1 successfully transformed ginsenoside rb1 into compound K [61]. Ginsenoside Rb1 and ginsenoside Rd were hydrolyzed into gypenoside XVII and compound K, respectively, by *Lactobacillus paralimentarius* LH4 [62]. Using the extracellular β-glucosidase of *Lactobacillus pentosus* DC10, compound K was produced from ginsenoside Rd [60].

For commercial applications, some researchers have utilized the strategy of whole-cell probiotic conversion with surface-displayed enzymes to hydrolyze glycosylated ginsenosides. When intracellular enzymes are collected using mechanical or chemical approaches, not only target enzymes, but also non-target metabolites can be extracted during the cell breakage process. In some cases, non-target metabolites can act as inhibitors for enzyme reactions [63]. Moreover, physicochemical treatments are likely to generate unwanted target-enzyme degradation. In addition, in order to collect and employ extracellular enzymes, time-consuming and labor intensive processes, such as protein separation, are necessary.

According to de Carvalho [64], the use of whole-cell catalysis is advantageous to save labor, production cost and/or maintenance costs. Furthermore, this process eliminates undesirable reactions generated by intracellular cell metabolites. Park *et al.* [65] transformed ginsenosides Rb1, Rc, Rd and F2 into compound K using whole cell *Leuconostoc mesenteroides* KFRI 690, *Leuconostoc paramesenteroides* KFRI 159 and *Lactobacillus delbrueckii* KCCM 35486. Both whole cell and disrupted cell homogenates of *Bifidobacterium longum* RD47 (Rd47) were utilized to hydrolyze ginsenoside Rb2 and Rc into ginsenoside Rd [22]. In their work, both α-l-arabinopyranosidase and α-l-arabinofuranosidase were detected from Rd47 and expressed notable resistance against sonication process. Similar work was done by both whole cell and disrupted cell homogenates of *Lactobacillus delbrueckii* Rh2 to produce baicalein and wogonin from baicalin and wogonoside [66]. They also employed whole cell β-glucosidase of *Lactobacillus rhamnosus* GG to transform ginsenoside Rb1 to Rd [67].

## 7. The Use of Modified MRS for Enzyme Production

Many researchers have attempted to culture probiotic bacteria in conventional MRS media containing glucose as an enriched nutrient, the key carbon source and metabolic precursor for microbial germination [68,69]. Generally, in the food and dairy industry, manufacturers have tried to enhance cell biomass productivity during the fermentation process to follow company guidelines and federal regulations. For instance, The National Yogurt Association has recommended a standard for commercial yogurts (e.g., at least 10^7^ CFU/g of active cultures, pH 4.6 or lower, the use of safe and suitable sweeteners, *etc.*) [70]. To achieve their goal, manufacturers generally use simple sugar or sucrose as a major ingredient to reduce cost and increase microbial nutrient supply. For bench, lab and pilot-scale experiments, multiple in-house and commercially-available media (*i.e*., LBS (Lactobacillus selective) and MRS) containing glucose have been used for *Lactobacillus* and *Bifidobacterium* spp. enrichment.

However, if the goal is to enhance the productivity of glycoside hydrolase from probiotic bacteria rather than enhancing the cell biomass productivity, then high levels of glucose can significantly inhibit glycosidase production [22,67,71,72]. During the cell enrichment process, media ingredients become enzyme substrates or substrate analogs, acting as significant enzyme inducers [73]. Specifically, when some media ingredients use sugar conjugates, such as disaccharides, oligosaccharide and polysaccharide, as a source of carbon, some probiotics produce glycosylases to effectively intake glucose after hydrolysis [35,74].

There are some examples that used composition-dependent systems, which introduce multiple carbon sources in the media and/or glucose-limited culture conditions. A recent report showed that *Lactobacillus rhamnosus* GG cultured in modified MRS containing cellobiose instead of glucose had 25-times higher β-glucosidase productivity compared to those cultured in conventional MRS [67]. The productivity of α-l-arabinofuranosidase and α-l-arabinopyranosidase of *Bifidobacterium longum* RD47 were significantly increased by additional supplements of 2% (*w*/*v*) ascorbic acid in MRS media [22]. In these works, the enhanced glucose or other sugar contents in the media resulted in significantly decreased activities of β-glucosidase, α-l-arabinofuranosidase and α-l-arabinopyranosidase from *Lactobacillus rhamnosus* GG and *Bifidobacterium longum* Rd47 compared to those cultured at controlled media conditions. This *Bifidobacterium longum* Rd47 was also cultured in in-house media containing soybean oligosaccharides instead of glucose and showed increased α- and β-galactosidase productivities [75]. *Lactobacillus delbrueckii* Rh2, cultured in MRS containing galactose instead of glucose, showed increased β-glucuronidase activity [66].

**Figure 2:**
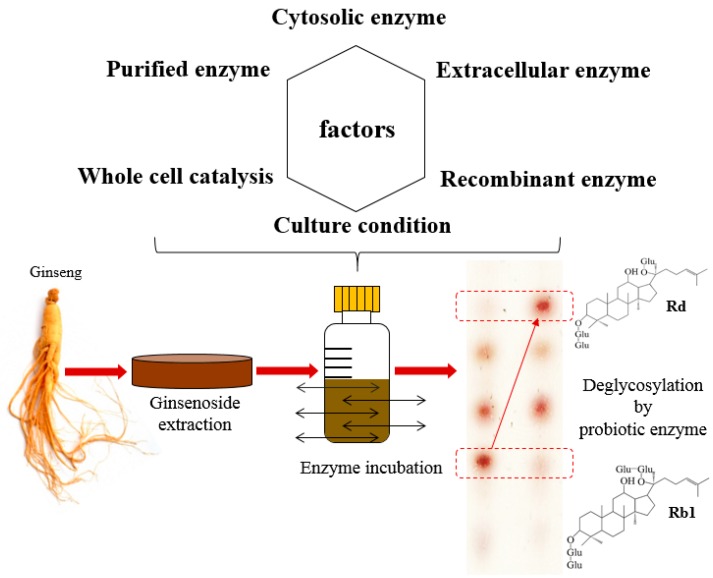
Application of probiotic enzymes prepared by multiple methods for the biotransformation of ginsenoside glycosides. The TLC profile was adapted and modified from Ku *et al.* [67].

Commercial application of whole cell enzymes from probiotic bacteria is still limited mainly due to a lack of methods, expertise and information about probiotic enzymes and their regulation. Therefore, further employing of controlled culture conditions using unconventional carbon sources and/or glucose-limited media is likely necessary to investigate microbial potential for certain enzyme production. An overall summary of enzyme preparation and its use for biocatalysis of glycosidated ginsenosides is shown in Figure 2.

## 8. Conclusions

Multiple structurally-similar ginsenosides with diverse numbers and locations of hydroxyl groups exhibit significantly different bioactive properties; specifically, ginsenoside glycosides have shown poor absorption rates in the body. In this regard, intentional biocatalysis of ginsenosides using probiotic enzymes is of significant industrial and scientific interest for the purpose of enhancing and standardizing their nutraceutical properties. Structural modification of glycosylated ginsenosides has been successfully achieved with higher productivity by ginsenoside aglycones. In the pharmaceutical and nutraceutical industry, there is a great potential for the use of genetically-engineered and conventionally-screened probiotic bacteria and their enzymes for the production of ginsenoside aglycones.

Although various probiotic enzymes have already been used for large-scale applications, various challenges of enzyme preparation from probiotic microorganisms possibly exist, including limited biomass productivity, uneconomical bioreaction and bioseparation and demanding rapid identification and screening systems. The enhanced efficacy of probiotic enzymes still needs to be adapted to the special needs of manufacturing processes. Recent reports also show that it is possible to use modified MRS to increase microbial enzyme productivities. These attempts have been made to enhance the productivity of glycosylases, which are applicable for the biotransformation of glycosylated ginsenosides. However, further improvements are necessary, including: (i) exploiting broad food-grade hosts for transformation; (ii) enhancing cell biomass and enzyme productivity; (iii) utilizing and determining the optimal media compositions for enzyme induction.

## Figures and Tables

**Table 1:** Examples of the ginsenosides present in ginseng and their chemical names. Adapted and modified from Chi *et al.* [17,18].

Ginsenosides	Chemical Names
Rb1	3-*O*-[b-d-glucopyranosyl-(1-2)-b-d-glucopyranosyl]-20-*O*-[b-d-glucopyranosyl-(1-6)-b-d-glucopyranosyl]-20(S)-protopanaxadiol
Rb2	3-*O*-[b-d-glucopyranosyl-(1-2)-b-d-glucopyranosyl]-20-*O*-[a-l-arabinopyranosyl-(1-6)-b-d-glucopyranosyl]-20(*S*)-protopanaxadiol
Rc	3-*O*-[b-d-glucopyranosyl-(1-2)-b-d-glucopyranosyl]-20-*O*-[a-l-arabinofuranosyl-(1-6)-b-d-glucopyranosyl]-20(*S*)-protopanaxadiol
Rd	3-*O*-[b-d-Glucopyranosyl-(1-2)-b-d-glucopyranosyl]-20-*O*-b-d-glucopyranosyl-20(*S*)-protopanaxadiol
Re	6-*O*-[a-l-rhamnopyranosyl-(1-2)-b-d-glucopyranosyl]-20-*O*-b-d-glucopyranosyl-20(*S*)-protopanaxatriol
Rg1	6-*O*-b-d-glucopyranosyl-20-*O*-b-d-glucopyranosyl-20(*S*)-protopanaxatriol
Rg2	6-*O*-[a-l-rhamnopyranosyl-(1-2)-b-d-glucopyranosyl]-20(*S*)-protopanaxatriol
Rh1	6-*O*-b-d-glucopyranosyl-20(*S*)-protopanaxatriol
Rh2	3-*O*-b-d-glucopyranosyl-20(*S*)-protopanaxadiol
F1	20-*O*-b-d-glucopyranosyl-20(*S*)-protopanaxatriol
F2	3-*O*-b-d-glucopyranosyl-20-*O*-b-d-glucopyranosyl-20(*S*)-protopanaxadiol
Compound K	20-*O*-b-d-glucopyranosyl-20(*S*)-protopanaxadiol
Compound O	3-*O*-b-d-glucopyranosyl-20-*O*-[a-l-arabinopyranosyl-(1-6)-b-d-glucopyranosyl]-20(*S*)-protopanaxadiol
Compound Y	20-*O*-[a-l-arabinopyranosyl-(1-6)-b-d-glucopyranosyl]-20(*S*)-protopanaxadiol
Mc	20-*O*-[a-l-arabinofuranosyl-(1-6)-b-d-glucopyranosyl]-20(*S*)-protopanaxadiol
